# Identification and Characterization of Novel Mutations in the Human Gene Encoding the Catalytic Subunit Calpha of Protein Kinase A (PKA)

**DOI:** 10.1371/journal.pone.0034838

**Published:** 2012-04-13

**Authors:** Kristoffer Søberg, Anja C. V. Larsen, Mandy Diskar, Paul H. Backe, Magnar Bjørås, Tore Jahnsen, Jon K. Laerdahl, Torbjørn Rognes, Friedrich W. Herberg, Bjørn S. Skålhegg

**Affiliations:** 1 Department of Nutrition, Institute of Basic Medical Sciences, University of Oslo, Oslo, Norway; 2 Department of Biochemistry, Institute of Basic Medical Sciences, University of Oslo, Oslo, Norway; 3 Department of Biochemistry, University of Kassel, Kassel, Germany; 4 Centre for Molecular Biology and Neuroscience (CMBN), Department of Microbiology, Rikshospitalet, Oslo University Hospital, Oslo, Norway; 5 Department of Medical Biochemistry, University of Oslo, Oslo, Norway; 6 Bioinformatics Core Facility, Department of Informatics, University of Oslo, Oslo, Norway; 7 Department of Informatics, University of Oslo, Oslo, Norway; Bioinformatics Institute, Singapore

## Abstract

The genes *PRKACA* and *PRKACB* encode the principal catalytic (C) subunits of protein kinase A (PKA) Cα and Cβ, respectively. Cα is expressed in all eukaryotic tissues examined and studies of Cα knockout mice demonstrate a crucial role for Cα in normal physiology. We have sequenced exon 2 through 10 of *PRKACA* from the genome of 498 Norwegian donors and extracted information about *PRKACA* mutations from public databases. We identified four interesting nonsynonymous point mutations, Arg45Gln, Ser109Pro, Gly186Val, and Ser263Cys, in the Cα1 splice variant of the kinase. Cα variants harboring the different amino acid mutations were analyzed for kinase activity and regulatory (R) subunit binding. Whereas mutation of residues 45 and 263 did not alter catalytic activity or R subunit binding, mutation of Ser_109_ significantly reduced kinase activity while R subunit binding was unaltered. Mutation of Cα Gly_186_ completely abrogated kinase activity and PKA type I but not type II holoenzyme formation. Gly_186_ is located in the highly conserved DFG motif of Cα and mutation of this residue to Val was predicted to result in loss of binding of ATP and Mg^2+^, which may explain the kinetic inactivity. We hypothesize that individuals born with mutations of Ser_109_ or Gly_186_ may be faced with abnormal development and possibly severe disease.

## Introduction

Protein Kinase A (PKA) is a cyclic AMP (cAMP)-dependent Ser/Thr kinase involved in regulating a multitude of biological processes including cell growth and division, cell differentiation, as well as metabolism and immune responsiveness [1]. In its inactive state, PKA exists as a tetrameric holoenzyme consisting of two regulatory (R) and two catalytic (C) subunits. Four different genes, *PRKAR1A*, *PRKAR1B*, *PRKAR2A*, and *PRKAR2B*, encode the R subunit proteins RIα, RIβ, RIIα, and RIIβ, respectively, with several splice variants of the RIα and RIIα subunits [2], [3]. Two principal genes, *PRKACA* and *PRKACB*, encode the C subunits Cα and Cβ, respectively. Also, *PRKX* and the retroposons *PRKY* and *PRKACG* are identified as PKA C subunit genes. While no protein products for *PRKY* and *PRKACG* have been identified, *PRKX* is translated into a protein kinase which is inhibited by the R subunit in a cAMP-sensitive manner [4]–[7]. Both the *PRKACA* and *PRKACB* genes encode several protein products. Alternative use of two exons upstream of exon 2 in the *PRKACA* gene gives rise to two Cα variants with different N-termini. These proteins are designated Cα1 and Cα2 [8]–[11]. In the case of the *PRKACB* gene, several exons 5′ of exon 2 are encoding a number of Cβ splice variants designated Cβ1, Cβ2, Cβ3, and Cβ4 [12], [13], as well as a number of Cβ3 and Cβ4 forms that contain N-terminal inserts from exons designated a, b, and c [14], [15]. In the brain and nervous tissues of higher primates, a range of C variants lacking exon 4 encoded sequences are also identified [16].

The two major groups of the R subunits RI and RII form two types of the PKA holoenzymes termed PKA type I and PKA type II, respectively. The C subunits are the catalytically active components, and they become activated after dissociation from the R subunits in a cAMP-dependent manner. For specificity in the cAMP-PKA signaling pathway, the PKA holoenzymes can be located to specific subcellular structures via A Kinase Anchoring Proteins (AKAPs). AKAPs typically bind RII subunits with high affinity [8], [17]–[19]. In contrast, PKA type I holoenzymes tend to locate to the soluble fraction of the cell but can bind to dual- and RI-specific AKAPs [19]. Both the C and R subunits are differentially expressed in various tissues. Whereas Cα1 is ubiquitously expressed, Cα2 is exclusively found in sperm cells [8]–[10]. For the Cβ variants, Cβ1 is ubiquitously expressed, while the other Cβ variants are more specifically expressed in certain tissues, such as lymphoid and neuronal tissues [14], [15].

Cα1 is the principal source of PKA activity in most tissues [11] and was the first protein kinase to be subjected to crystallization and 3D structure determination [20]. The Cα1 structure reveals a large C terminal lobe (C-lobe or large lobe) and a smaller N terminal lobe (N-lobe or small lobe). The large lobe is mainly composed of α-helices, and is involved in R subunit and substrate binding. It contains a number of residues involved in catalysis. The small lobe is mostly composed of β-strands and contains an ATP binding site. Between the large and small lobe is the active site cleft. The small and large lobes, as well as the active site, are known as the catalytic core and are encoded by residues 40–300 in PKA Cα1. The amino acid sequence and 3D structure of the catalytic core are conserved among all protein kinases. The catalytic core may, in addition to ATP, bind two Mg^2+^ ions that are critical for catalysis [20]. The two Mg^2+^ ions are designated activating and inhibitory Mg^2+^, or Mg1 and Mg2, respectively [21]. The binding affinity for Mg1 is higher than for Mg2 in the presence of ATP, while binding of Mg2 is believed to stabilize the protein, but also to inhibit catalysis [21]–[24]. Mg^2+^ is also essential for high-affinity binding to inhibitors PKI and RIα subunits [25]. For catalytic activity, Cα has to be phosphorylated on residue Thr_197_, located in the activation loop of the large lobe. This phosphorylation affects the conformation of the conserved DFG (Asp_184_-Phe-Gly) motif in the active site [26]. In the active conformation, Asp_184_ of the DFG motif coordinates the three phosphates of ATP via the Mg^2+^ ions, positioning the γ-phosphate of ATP optimally for catalysis. This central role of Asp_184_ in catalysis depends on a conserved hydrogen bond between Asp_184_ and Gly_186_ backbone amide group. This interaction orients Asp_184_ perfectly for coordination of the Mg^2+^ ions and efficient ATP binding [27]. The Asp_184_-Gly_186_ hydrogen bond is only established after phosphorylation of Thr_197_, which causes a twist of the peptide bond between Phe_185_ and Gly_186_. In this way phosphorylation of Thr_197_ serves as a regulatory mechanism for the activation of the C subunit. The Local Spatial Pattern (LSP) alignment method, developed by Kornev et al [27], has revealed two conserved spatial motifs in eukaryotic protein kinases. These are two hydrophobic structures called the Catalytic (C-) and Regulatory (R-) spines that have to be established in order for the kinase to become catalytically active. The C-spine is completed when the adenine nucleobase of ATP interacts with the active site. For the R-spine to be established, Thr_197_ in the activation loop has to be phosphorylated. Evaluation of the C- and R-spines is a helpful way of predicting whether a kinase has a catalytically active or inactive conformation.

Despite its central role in a multitude of processes in the body, few diseases have been linked to defects or alterations in the PKA subunit genes or proteins. Germline mutations leading to premature stop codons in *PRKAR1A* have been identified in patients with Carney complex, a multiple neoplasia syndrome with skin pigmentations, cardiac and other myxomas, endocrine tumors, and psammomatous melanotic schwannomas [28], [29]. To the best of our knowledge, no diseases have been linked to defects in any of the C subunit genes. Nevertheless, studies on mice that are homozygote knockout (KO) for the *PRKACA* gene reveal a severe phenotype. The majority of the Cα^−/−^ mice die at birth or during the early postnatal period [30]. The offspring that survive beyond 2 months (< 11%, [31]), all show reduced growth, and the few males, but not females, that reach puberty are all 100% infertile [30], [32]. Furthermore, mice with reduced PKA C gene transcription by only expressing one functional C subunit allele of Cα or Cβ, show reduced PKA activity and neural tube defects [33].

Due to the severe phenotype of Cα KO mice we searched for mutations in the human *PRKACA* gene by genomic sequencing of 498 subjects. We identified two nonsynonymous point mutations in the *PRKACA* gene that result in amino acid switches in the Cα1 protein at residues 45 and 109. In addition, we searched for previously described nonsynonymous mutations in various human genomic DNA databases, and selected two of these, giving amino acid switches at residues 186 and 263, for further studies. These four mutations were introduced to Cα1- and Cα2-encoding plasmids and the proteins were expressed and analyzed with respect to kinase activities and R subunit binding *in vitro* and *in vivo*. Mutation of residues 109 and 186 were associated with significantly reduced and totally abrogated kinase activity, respectively. In addition, mutation of residue 186 rendered Cα incapable of forming PKA type I holoenzymes.

## Materials and Methods

### Analysis of Mutations

The *PRKACA* gene from 498 Norwegian donors deriving from three different control groups [34], [35] was sequenced and analyzed for point mutations by Lark Technologies (Takeley, UK). Leukocyte DNA was isolated from thawed blood containing ethylenediaminetetraacetic acid. Using the Applied Biosystems 340A Nucleic Acid Extractor, DNA was extracted with chloroform/phenol followed by ethanol precipitation. Exons 2–10 of *PRKACA* were sequenced using the primers listed in table 1.

**Table 1:** Primers used for sequencing of *PRKACA* exons 2 to 10.

Sequencing of exon no.	Primers
2	Forward: 5′-AGGCTCTGGGTTGGAACTGC-3′
2	Reverse: 5′-CTGGCATTGGGCAGTCAGGT-3′
3	Forward: 5′-GCCTTAAGGAATGTGCCCTC-3′
3	Reverse: 5′-TGGCGAAAACCCATCTCTAC-3′
4	Forward: 5′-CCACGGCTCTGACCTCTGTG-3′
4	Reverse: 5′-GATGTTACTGAGGTTGGGTG-3′
5	Forward: 5′-CTCAAGCTCCCAGAGCAAGG-3′
5	Reverse: 5′-TACTGGTGAGAACCACCGTG-3′
6–8	Forward: 5′-AATGCAGCCACATTGTTGAG-3′
6–8	Reverse: 5′-TTGAGGTGTTGGCCTCAGTG-3′
9	Forward: 5′-ACGTAGTGAGACCCTGTCTC-3′
9	Reverse: 5′-CGAAACTCTTAATGTAGCAACTC-3′
10	Forward: 5′-AGTGGTTTGCCACAACTGAC-3′
10	Reverse: 5′-AGCTGGTGTTTCTGTCCCTC-3′

A search for mutations in the *PRKACA* gene was also performed in the following public databases: NCBÍs dbSNP [36], [37], Ensembl [38], and SNPper [39].

### Generation of Plasmids

The pDONR201-Cα1_WT_ vector has previously been described [40] and was used to make plasmids containing Cα1_Mut_ using QuikChange® II Site-Directed Mutagenesis Kit (Stratagene, cat. no. 200524) according to the manufacturer’s protocol. The pDONR201-Cα1 variants were transferred to the eukaryotic expression vector pEF-DEST51 by the LR recombination reaction (Invitrogen, cat. no. 11791–019) according to the manufacturer’s protocol.

Cα1_WT_ and Cα2_WT_ prokaryotic expression vectors are previously described [41]. Using QuikChange^TM^ Site-Directed Mutagenesis Kit (Stratagene, cat. no. 200518), Cα2_WT_ was mutated into Cα2_Gly186Val_.

Cα1 was cloned into the mammalian expression vector pGFP^2^-C3 (Perkin Elmer) as previously described [42], creating the BRET sensor construct GFP-Cα1_WT_. Using Quikchange® II Site-Directed Mutagenesis Kit, GFP-Cα1_WT_ was mutated into GFP-Cα1_Mut_, according to the manufacturer’s protocol. RIα-Rluc and RIIα-Rluc constructs have previously been described [42].

For all mutagenesis reactions, the PCR reaction mixture was initially heated to 95°C for 30 s, followed by 16 cycles of 95°C for 30 s, 55°C for 1 min and 68°C for 7 min, before final elongation at 68°C for 2 min. Primers are listed in table 2. All plasmids were verified by sequencing (Eurofins MWG Operon).

**Table 2:** Primers for mutagenesis (mutated nucleotide in bold).

Desired mutation	Primers
Arg45Gln	Forward: 5′- CTTGGATCAGTTTGAAC**A**AATCAAGACCCTCGGC-3′
Arg45Gln	Reverse: 5′-GCCGAGGGTCTTGATT**T**GTTCAAACTGATCCAAG-3′
Ser109Pro	Forward: 5′-CGTCAAACTCGAGTTC**C**CCTTCAAGGACAACTC-3′
Ser109Pro	Reverse: 5′-GAGTTGTCCTTGAAGG**G**GAACTCGAGTTTGACG-3′
Gly186Val	Forward: 5′-GGTGACAGACTTCG**T**TTTCGCCAAGCGCG-3′
Gly186Val	Reverse: 5′-CGCGCTTGGCGAAA**A**CGAAGTCTGTCACC-3′
Ser263Cys	Forward: 5′-CCTTCCCACTTCAGCT**G**TGACTTGAAGGACC-3′
Ser263Cys	Reverse: 5′-GGTCCTTCAAGTCA**C**AGCTGAAGTGGGAAGG-3′

### Purification of Proteins

Cα1_WT_ and Cα2_WT_ were purified by affinity chromatography using PKI-peptide Affi-Gel, as previously described [41]. Cα2_Gly186Val_ was purified using a modified method described by Hemmer et al [43]. Briefly, BL21(DE3) cells transformed with either Cα2_Gly186Val_ or His-tagged RIIα_Gly337Glu_ (provided by Antje Badel, University of Kassel, Germany) were cultured and protein expression induced with IPTG. After centrifugation, the bacterial pellets were resuspended and lysed, and the lysates were mixed in equimolar amounts forming PKA holoenzymes. The holoenzymes were then coupled to a Ni^2+^-resin binding the His-tagged R subunits. Following washing with 50 mM NaH_2_PO_4_ (pH 8.0), 5 mM β-mercaptoethanol, and 25 mM KCl, Cα2_Gly186Val_ was eluted with the same buffer supplemented with 10 mM cAMP.

### Cell Cultures and Transfection Methods

HEK 293T cells (ATTC, cat. no. CRL-11268) were cultured at 37°C in humidified air with 5% CO_2_ in RPMI 1640 (Sigma-Aldrich, cat. no. R0883) supplemented with 10% fetal bovine serum (Sigma-Aldrich, cat. no. F7524), 1% non-essential amino acids (GibcoBRL, cat. no. 11140–035), 1% L-glutamine (Sigma-Aldrich, cat. no. G7513), sodium pyruvate (GibcoBRL, cat. no. 11360–039) and 1% Penicillin-Streptomycin solution (Sigma-Aldrich, cat. no. P4458). The 293T cells were transfected with Lipofectamine 2000 (Invitrogen, cat. no. 11668–019) according to the manufacturer’s instructions.

COS-7 cells (ATCC, cat. no. CRL-1651) were cultivated in Dulbecco’s modified Eagle’s medium (DMEM, Sigma-Aldrich), with 10% (v/v) fetal calf serum (FCSgold, PAA, Coelbe, Germany) at 37°C and 5% CO_2_. Cells were split in a 1∶3 ratio every 3–4 days and transfected using PEi (Polysciences, 25 kDa linear polyethylenimine) [44]. Cells were transfected as previously described [42]. Briefly, 2×10^4^ COS-7 cells were seeded in each well of a 96-well microplate (Nunclon Surface, cat. no. 136101). After 24 h, cells were transfected with plasmid DNA (0,2 µg per well) using PEi.

### Phosphotransferase Assay

20 h post transfection, the HEK 293T cells were harvested and washed 3 x in phosphate buffered saline, then lysed in a potassium-phosphate buffer (5 mM K_2_HPO_4_, 1 mM EDTA, 250 mM sucrose and 0.5% Triton X-100, 1 mM phenylmethanesulfonyl fluoride, 1 mM Na_3_VO_4_ and protease inhibitor cocktail (Sigma-Aldrich, cat. no. P8340)) by vortexing and 20 min on ice. Debris was pelleted by centrifugation at 16 000 g for 15 min at 4°C. After Bradford protein determination (Bio-Rad, cat. no. 500–0006), all samples were adjusted to equal concentrations. Phosphotransferase activities of lysates from Cα1_WT_, Cα1_Mut_, and mock transfected cells were determined by the phosphotransferase assay described by Witt and Roskoski [45], [46]. Briefly, phosphotransferase activity against the PKA-specific substrate Kemptide (Leu-Arg-Arg-Ala-Ser-Leu-Gly, Sigma-Aldrich) was measured in a reaction mixture (14.3 mM Mg-acetate, 143 µM ATP, 7.5 µCi/mL γ^−32^P-ATP (PerkinElmer), 50 mM Tris-HCl, pH 7.4) in the presence or absence of cAMP or PKI. After incubation at 30°C for 9 min, the reactions were stopped by spotting onto p81 phosphocellulose papers followed by 4 × 15 min washing in 75 mM phosphorous acid. The filter papers were washed in 96% ethanol for 10 min and air dryed for approximately 1 h. Phosphotransferase activity was measured by liquid scintillation in 3 ml Opti-fluor (Packard BioScience, PerkinElmer). All experiments were repeated at least three times.

### BRET Assay

48 h post transfection cells were washed with 1 × PBS and BRET read-out was performed by addition of 5 µM of the luciferase substrate coelenterazine 400 a (Biotrend, Cologne, Germany). After simultaneous detection of RLuc (410 nm) and GFP^2^ (515 nm) emission by a multi-label reader (POLARstar, Omega, BMG Labtech GmbH, Ortenberg, Germany) the ratiometric BRET signal was calculated as followed [emission_515 nm_ - (not transfected) COS-7 cells_515 nm_]/[emission_410 nm_- (not transfected) COS-7 cells_410 nm_] using the GraphPad Prism 4 software (La Jolla, CA, USA). Experiments were performed both in absence and presence of 50 µM forskolin (fsk) and 100 µM 3-isobutyl-1-methylxanthine (IBMX) (Sigma-Aldrich). All experiments were repeated at least three times.

### Immunoblotting

Samples used in phosphotransferase assays were evaluated for C subunit expression. Protein concentrations of each sample within an assay were adjusted to the same levels, as described above. Proteins were separated by SDS-PAGE and transferred to PVDF membranes. The membranes were blocked by drying and rehydrated in methanol before incubation with primary antibody Purified Mouse Anti-PKA [C] (1∶250 dilution, BD Transduction Laboratories, cat. no. 610981) for 1 h, followed by washing with TBST. After 30 min incubation with secondary antibody HRP conjugated goat anti-mouse (1∶2000 dilution, ICN Biomed, cat. no. 55563) and washing, proteins were detected by enhanced chemiluminescence (Pierce Biotechnology, Rockford, IL, USA) and the Syngene G:BOX imaging system.

Expression of endogenous C and GFP-C constructs in transfected COS-7 cells used for BRET assays were also assessed with immunoblotting. Proteins were detected similarly to the method described above, with the following modifications: blocking was performed with 5% milk powder for 1 h; primary antibody was rabbit anti-Cα (Santa Cruz, PKA cat (c-20) cat. no. SC-903) and secondary antibody anti-rabbit IgG (peroxidase-linked species-specific whole antibody (ECL) NA934, GE Healthcare, Freiburg, Germany).

### Spectrophotometric Kinase Activity Assay

Kinetic activities of purified Cα2_WT_ and Cα2_Gly186Val_ were evaluated by the continuous enzyme-linked spectrophotometric method described by Cook et al. [22]. Activities were reported in U/mg, defined as µmolmg^−1^min^−1^. All experiments were repeated at least three times.

### Molecular Representation and Simulation

Selected motifs, including the DFG motif and it’s interactions with ATP and divalent cations were presented and analyzed with PyMOL [47], using the experimental structure described by Thompson et al [48] (PDB identifier 3FJQ). Simulated mutagenesis in PyMOL was performed on Gly_186_ in the DFG motif. Relevant distances between the Gly_186_/Val_186_ residue and surrounding atoms were calculated.

## Results

**Figure 1:**
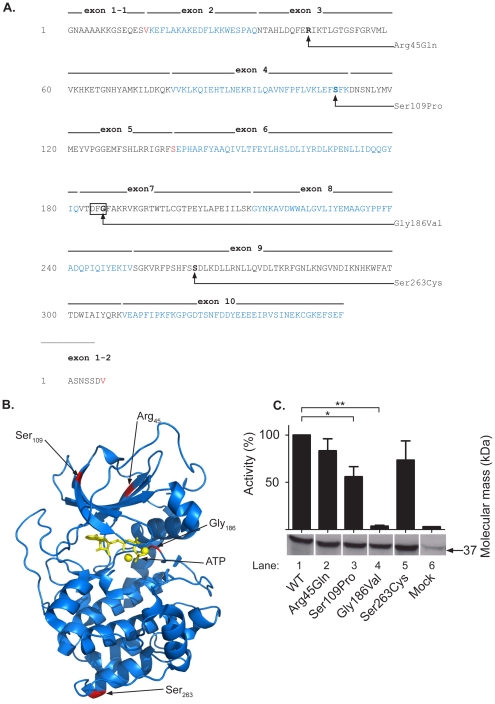
Sequences of human wild type PKA Cα1/Cα2, location of point mutations, and activities of mutant proteins. **A.** The human Cα wild type and mutated amino acid sequence. The N-terminal methionine of Cα1 is removed post-translationally, and glycine residue 1 in mature Cα1 corresponds to codon 2. Alternating black and blue coloring shows the contribution of the eleven exons to the mature translated protein. The codons for Val_15_ and Ser_139_ (red) are encoded by nucleotides from neighboring exons. The *PRKACA* gene encodes two splice variants, Cα1 and Cα2 (UniProt [59] accession number P17612), which differs by alternative use of exons 1–1 or 1–2, respectively. The four point mutations are highlighted and denoted with the amino acid change. The Gly186Val mutation is located in the DFG motif (boxed) [26]. **B.** The location of the four mutations are indicated (red) in a model of Cα1 based on the PDB structure 3FJQ [48]. Residues are designated according to the WT Cα1 sequence. ATP and two divalent cations are shown in yellow. **C.** Immunoblotting and phosphotransferase assays of WT and mutated Cα1 subunits. Cα1 was expressed in HEK 293T cells and immunoreactive C subunit detected with anti-C (Anti-mouse PKA[C], cat. no. 610981) after separation of cell extracts by SDS-PAGE in 10% gels and immunoblotting. Expression of Cα1_WT_ is shown in lane 1 (WT), mutated Cα1 in lanes 2 to 5, and endogenous Cα1 in lane 6 (Mock). Activities are normalized relative to Cα1_WT_ activity. Data represents mean values ± standard deviation (SD) of triplicate experiments (*, P < 0.05. **, P < 0.005).

We performed two independent studies of the *PRKACA* gene; *1)* by sequencing genomic DNA from 498 Norwegian subjects and *2)* by a bioinformatics analysis of DNA sequences from various populations submitted to publicly available databases (see Material and Methods). By the first approach, exons 2 through 10 of *PRKACA* were sequenced using exon-specific primers and genomic DNA extracted from leukocytes. In this way we detected five mutations in the *PRKACA* gene; one in each of exons 2, 3, 4, 5, and 8. Only two of the mutations translated into an amino acid switch. The corresponding nucleotides were located in exons 3 and 4, and are affecting Arg_45_ and Ser_109_ in the Cα1 sequence, while the three silent mutations were at Pro_33_, Gly_126_, and Pro_236_ (Table 3, Fig. 1A). With the recent submission of a large amount of new mutations from several thousand individuals by exome and whole genome sequencing projects, four of these five mutations are now also present in dbSNP [36], [49]. Our study thus confirms the presence of the mutations referred to as rs56105247 (at Pro_33_), rs56085217 (Arg_45_), rs78098302 (Gly_126_), and rs137911238 (Pro_236_) in the Norwegian population.

**Table 3:** Mutations in the *PRKACA* gene discovered by sequencing of 498 Norwegian subjects.

Exon no.	Nucleotide (nt) no.	Amino acid (aa)	WT/mut nt	WT/mut aa	No. affected individuals	Functional consequence prediction
2	102	Pro33	C/T	Silent	10	Likely neutral
3	137	Arg45	G/A	Arg/Gln	1	Arg45 is highly conserved in metazoan Cα/Cβ homologs – likely functional consequences
4	328	Ser109	T/C	Ser/Pro	2	Ser109 in conserved in Cα, but not in Cβ – effect unknown
5	381	Gly126	G/A	Silent	3	Likely neutral
8	711	Pro236	G/A	Silent	1	Likely neutral

Nucleotide enumeration is counted from the PKA Cα1 transcript start codon. Amino acid numbering is given for the mature protein with N-terminal Gly_1_ corresponding to codon 2. Sequence conservation is illustrated in Supplementary Fig. S1.

By the second approach, we detected another twelve nonsynonymous mutations (Table 4), of which the majority has been submitted within the last year. Among the mutations that were known at the initiation of this study, two were selected for further investigations. Firstly, the mutation rs35635531, resulting in a switch of Ser_263_ to Cys, is the only mutation that has been submitted by three independent projects. Although the frequency of this mutation has not been determined in detail, it is likely to be relatively common in at least some human populations. Secondly, the mutation causing the Gly186Val mutation was considered particularly intriguing since this residue is located in the Mg^2+^ positioning loop close to ATP and Mg^2+^ in the active site cleft of Cα (Fig. 1B) and is part of the highly conserved DFG motif [26], [50], [51]. The positions of the four investigated mutations resulting in amino acid switches are shown relative to exon encoded sequence in Fig. 1A and in the 3D structure of Cα1 in Fig. 1B.

**Table 4:** Additional nonsynonymous mutations in the *PRKACA* gene found in public databases.

dbSNP	Exon no.	Nucleotide no.	Amino acid	WT/mut nt	WT/mut aa	Functional consequence prediction
rs142045517	2	103	Ala34	G/A	Ala/Thr	Ala34 is Ser in rodent Cα and Thr in human Cβ – likely few functional consequences
rs56029020	3	121	Leu40	T/G	Leu/Val	Leu40 is absolutely conserved in metazoan Cα/Cβ homologs – likely deleterious
rs112360106	3	149	Leu49	T/A	Leu/His	Conserved in metazoan Cα/Cβ homologs – likely deleterious
rs148280386	5	409	Gly136	G/A	Gly/Arg	Conserved in metazoan Cα/Cβ homologs – likely deleterious
rs142007512	6	541	Ile180	A/G	Ile/Val	Highly conserved in vertebrate Cα/Cβ – possibly functional effects
rs11541563	7	560	Gly186	G/T	Gly/Val	Conserved in metazoan Cα/Cβ homologs – likely deleterious
rs34988264	8	673	Leu224	-/G, 1 nt insertion		Frame shift in exon 8 – very likely results in nonfunctional Cα
rs35635531	9	791	Ser263	C/G	Ser/Cys	Highly conserved in metazoan Cα/Cβ homologs – possibly functional effects
rs146946205	9	842	Arg280	G/A	Arg/His	Conserved in metazoan Cα/Cβ homologs – likely deleterious
rs187770246	9	926	Arg308	G/A	Arg/Lys	Conserved as Arg or Lys in metazoa – likely few consequences
rs149832080	10	955	Phe318	T/C	Phe/Leu	Not conserved in vertebrates – likely few consequences
rs141087932	10	1045	Ser348	T/C	Ser/Pro	Not conserved in mammals – likely few consequences

Enumeration is given as in Table 3, and sequence conservation is illustrated in Supplementary Fig. S1. Reference identifiers from dbSNP [36], [49] are included in the left column.

### Mutation of Residues Ser_109_ and Gly_186_, but not Arg_45_ and Ser_263_ in Cα1 Influences Catalytic Activity

**Figure 2:**
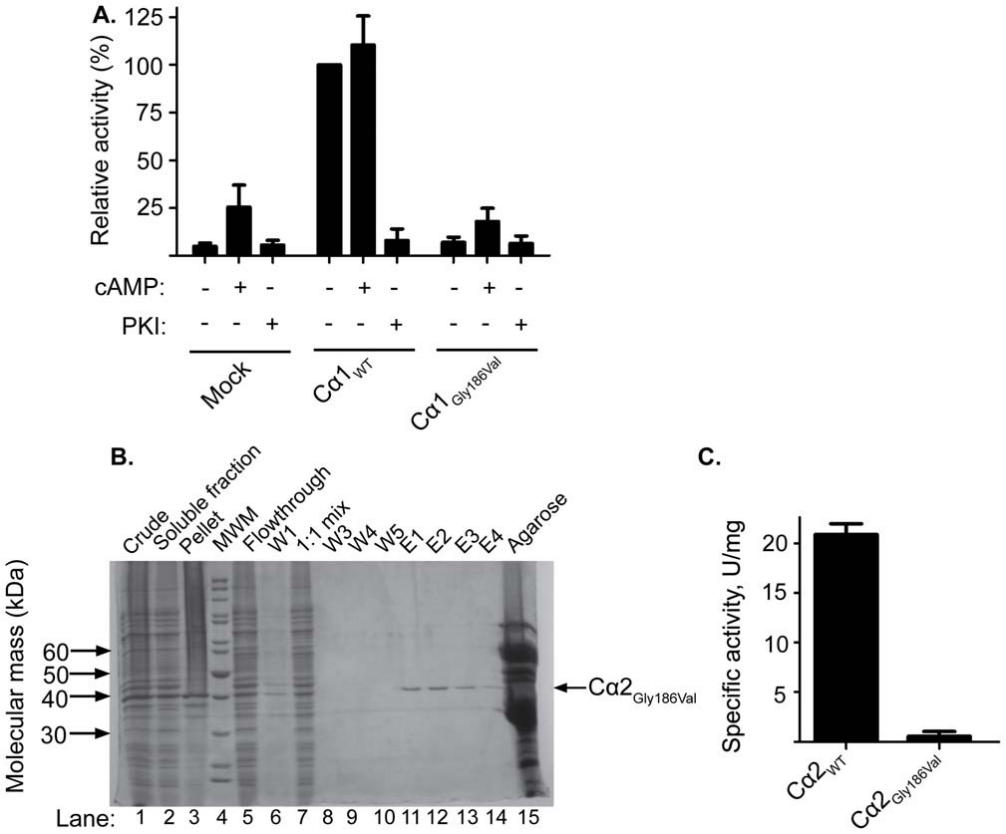
Activity of wild type and Gly186Val mutated Cα1 and Cα2. **A.** Phosphotransferase activity of Cα1_WT_ and Cα1_Gly186Val_ expressed in HEK-293T cells was measured in the presence of Kemptide and γ-^32^P-ATP, and in the presence (+) and absence (−) of cAMP or PKI kinase inhibitor. Enzyme activity was normalized according to activity of Cα1_WT_ which was set to 100%. The data are presented as mean values ± SD of triplicate experiments. **B.** Recombinant Cα2_WT_ (not shown) and Cα2_Gly186Val_ were expressed in BL21 (DE3) cells. For Cα2_Gly186Val_, expressed protein was purified by running cell extracts over a Ni-resin loaded with His6RIIα_Gly337Glu_ affinity column and eluted with 10 mM cAMP. The purification steps of Cα2_Gly186Val_ are shown after separation of the various fractions by SDS-PAGE. The crude cell extract (lane 1, crude) was centrifuged and divided into a soluble (lane 2) and an insoluble fraction (lane 3, pellet). Proteins not retained on the column is shown (lane 5, Flowthrough). W1, W3, W4 and W5 (lanes 6 and 8–10) depict protein contents of successive washing steps using buffer containing 50 mM NaH_2_PO_4_ (pH 8.0), 5 mM β-mercaptoethanol, and 25 mM KCl. E1-E4 (lanes 11–14 ) depict protein content in consecutive elution fractions using buffer containing 50 mM NaH_2_PO_4_ (pH 8,0), 10 mM cAMP, 5 mM β-mercaptoethanol, and 25 mM KCl. To assure equal amounts of RIIα_Gly337Glu_ and Cα2_Gly186Val_, cell extracts of RIIα_Gly337Glu_ and Cα2_Gly186Val_ expressed in separate bacteria cultures were mixed 1∶1 (lane 7, 1∶1 mix). Protein with the correct molecular mass (arrow to the right) was obtained in fractions E1–E3. Ten µl was applied in lanes 6, 8, 9 and 10, seven µl in lane 1, 2, 3, 5 and 7, and five µl in lane 4. (Molecular Weight Marker (MWM)). **C.** Kinase activity of recombinant Cα2_WT_ and Cα2_Gly186Val_ was determined by employing the spectrophotometric Cook assay. The data are presented as mean values ± SD of triplicate experiments.

We first tested if any of the mutations influenced the catalytic activity of Cα. Site-directed mutagenesis was used to introduce the required amino acid switches Arg45Gln, Ser109Pro, Gly186Val, and Ser263Cys to Cα encoding expression vectors. Wild type (WT) and the four mutated Cα1 products (collectively termed Cα1_Mut_) were expressed in HEK-293T cells, and 20 h post transfection the cells were lysed. Expression of protein was first assessed by immunoblotting with a pan C antibody, revealing that both Cα1_WT_ (Fig. 1C, lane 1) and Cα1_Mut_ (Fig. 1C, lanes 2 to 5) were expressed at comparable levels and with similar apparent size as endogenous Cα1 (Fig. 1C, lane 6). We further determined their catalytic activity in the presence of the PKA-specific substrate Kemptide and γ-^32^P-ATP (Fig. 1C). In these experiments, lysates from untransfected cells (Fig. 1C, lane 6) were included to assess endogenous kinase activity. This demonstrated high catalytic activity of expressed Cα1_WT_ which was set to 100% (Fig. 1C, lane 1). According to this assay, the catalytic activities of Cα1_Arg45Gln_ and Cα1_Ser263Cys_ were shown not to be significantly different from Cα1_WT_. On the other hand, the activity of Cα1_Ser109Pro_ was significantly lower compared to Cα1_WT_ (P <0.05), and the activity of Cα1_Gly186Val_ was comparable to background levels (Fig. 1C, lanes 4 and 6). This suggested that mutation of Gly_186_ rendered Cα inactive and prompted us to focus further on revealing the mechanism for this inactivation. Accordingly, we transfected cells with Cα1_Gly186Val_ and Cα1_WT_ and compared catalytic activity in the absence and presence of cAMP and the PKA-specific inhibitor PKI and compared phosphotransferase activities to mock transfected cells (Fig. 2A). The activity of Cα1_WT_ was reduced to background level in the presence of PKI while activities of Cα1_Gly186Val_ were comparable to background levels independently of stimulation with cAMP or inhibition with PKI (Fig. 2A, Mock compared to Cα1_Gly186Val_).

To determine if Cα_Gly186Val_ was completely inactive we next expressed and purified recombinant Cα_WT_ and Cα_Gly186Val_. We used recombinant WT and mutated Cα2 since Cα2 is more easily produced than Cα1 due to higher solubility [41]. Cα2 will therefore give a higher protein yield. Cα1 and Cα2 may also be interchanged in these experiments because studies suggest that they are kinetically indistinguishable [41]. WT and mutated recombinant Cα2 (Cα2_WT_ and Cα2_Gly186Val_) were produced in BL21(DE3) cells, purified and visualized by SDS-PAGE (Fig. 2B). (In order to simplify the nomenclature we refer to the mutations according to their Cα1 numbering also when introduced to Cα2. The actual position of the mutated residues in the Cα2 protein is achieved by subtracting the number 8). Cα2_Gly186Val_ was purified by running protein extracts over an RIIα_Gly337Glu_ affinity column and Cα2 bound to RIIα was eluted with 10 mM cAMP (see Material and Methods). The purification process was evaluated after each step by SDS-PAGE analysis (Fig. 2B, lanes 1 to 14). Purified Cα2_Gly186Val_ and Cα2_WT_ were tested for catalytic activity employing the Cook assay [22] that showed a specific activity for Cα2_WT_ at 20.9 ± 2.1 U/mg (95% confidence interval) (Fig. 2C, Cα2_WT_). In comparison, catalytic activity by Cα2_Gly186Val_ was undetectable demonstrating that a Gly_186_ to Val mutation renders the kinase catalytically inactive (95% confidence interval of Cα2_Gly186Val_: 0.55 ± 0.96 U/mg) (Fig. 2C, Cα2_Gly186Val_).

### Mutation Gly186Val Alters the Catalytic Core of Cα1

**Figure 3:**
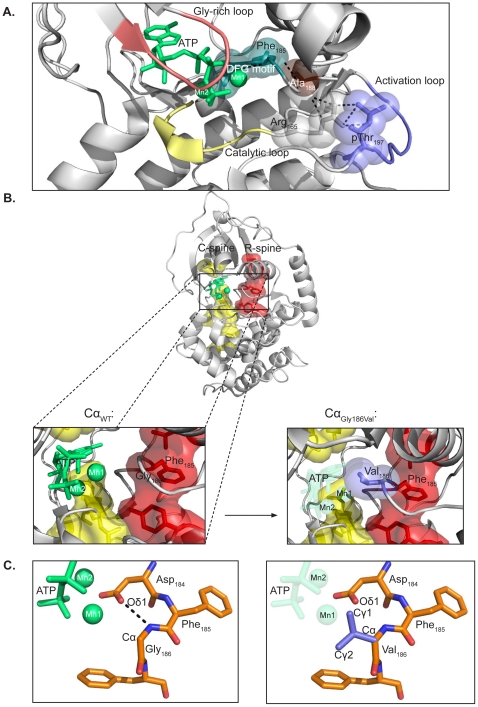
Mutation of Gly186 in Cα1 prevents Cα from binding ATP and divalent cations. ** A.** The 3D structure of the catalytic site of Cα1. Selected conserved motifs and their relations to divalent cations Mn1 and Mn2 and ATP are shown. Residues connecting phospho-Thr_197_ (pThr_197_) to the DFG motif are represented as stick models [50]. Mn_2_ATP (green), the DFG motif (teal), Gly-rich loop (salmon), catalytic loop (yellow), and activation loop (purple) are also highlighted. **B.** Overall structure of Cα1_WT_ with the conserved structural motifs the C- and R-spine structural motifs highlighted. The boxed segments depict spatial relations between residue 186 (Gly or Val) and ATP, divalent cations, and the C- and R-spines. **C.** DFG motif in Cα1_WT_ (left) and Cα1_Gly186Val_ (right) and its relations to Mn1 and ATP. Residues are represented as stick models with carbon (orange), oxygen (red) and nitrogen (blue) atoms. The hydrogen bond between the side chain of Asp_184_ and the amide group of Gly_186_ (dashed line) is predicted to be broken in Cα1_Gly186Val_ due to the Val side chain. The models are based on the structure with PDB identifier 3FJQ [48].

We next investigated the Gly186Val mutation in a 3D structure model of Cα1 in an attempt to understand the molecular nature of the kinetic inactivity (Fig. 3). Figure 3A shows selected conserved motifs surrounding the DFG motif where residue 186 is located. The chain of interactions leading into the DFG motif from phosphorylated Thr_197_ is depicted. In the active conformation of Cα1_WT_, the C- and R-spines are assembled and the structure is optimized for catalysis (Fig. 3B). Simulated mutagenesis of Gly186Val revealed three possible rotamers. For representation in the figures, the rotamer with the least steric hindrance was selected. Our Cα1_Gly186Val_ model suggests an altered conformation of the DFG motif, which we hypothesize may disturb the structure of the spines (Fig. 3B, lower right box). In all published experimental structures of catalytically active kinases, there is a conserved hydrogen bond between the side chain of Asp_184_ and the amide group of Gly_186_ [50] (Fig. 3C, left panel, dashed line). In figure 3C (right panel) it is shown that the presence of the hydrophobic Val side chain leads to steric hindrance and an unfavourable binding of the cation Mn1 (Cγ1-Mn1 distance 2.7 Å) compared to Gly (Cα-Mn1 distance 4.3 Å). The simulated distances between Cγ1, Cγ2 and Mn1 as well as ATP for this rotamer and the two other rotamers of Cα1_Gly186Val_ were reduced (results not shown). According to these data we hypothesize that by replacing Gly_186_ with Val, the bulky side chain of Val will make it less likely for Cα to bind Mg1/Mn1 in its optimal position. This will also prevent ATP binding, together suggesting why Cα2_Gly186Val_ lacks kinase activity.

A recent study on the Raf kinase where Gly_596_, which is the counterpart of Gly_186_ in Cα1, was mutated to Arg revealed that some Raf_Gly569Arg_ kinase activity was achieved at high concentrations of ATP [52]. Based on this study and considering the molecular structure depicted in figure 3 we speculated if increasing the concentrations of ATP and/or Mg^2+^ would be sufficient to restore at least some kinase activity in Cα2_Gly186Val_. Kinase activity of purified Cα2_Gly186Val_ and Cα1_WT_ were measured against increasing doses of Mg^2+^ and ATP, keeping either the ATP or Mg^2+^ concentration constant at 143 µM or 14.3 mM, respectively. For Cα1_WT_ high phosphotransferase activity was detected at concentrations of Mg^2+^ between 0.2 and 100 mM and ATP-concentrations between 1 and 143 µM. At none of these concentrations was activity of Cα2_Gly186Val_ detectable.

### Cα2_Gly186Val_ Associates with RIIa but not RIa

**Figure 4:**
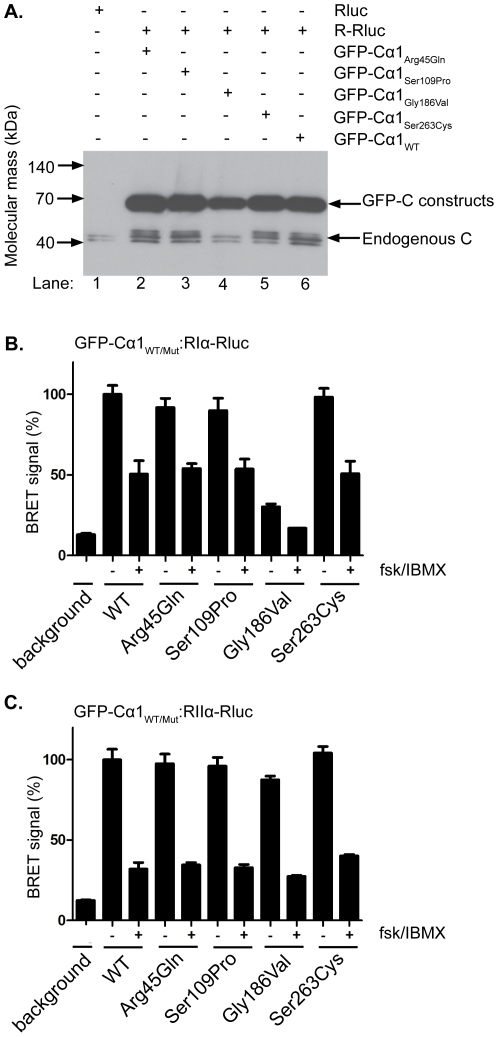
Assessment of PKA type I and II holoenzyme formation and dissociation with Cα_WT_ and Cα_Mut_ applying BRET in living cells. Co-expression of GFP-Cα1_WT_ or GFP-Cα1_Mut_ with wt RIα and RIIα subunits fused to *Renilla* luciferase (RIα-Rluc, RIIα-Rluc, as described in Diskar et al [55] and Material and Methods). **A.** Western blot analysis of COS-7 cells expressing either *Renilla* luciferase alone (lane 1), or R-Rluc plus GFP-Cα1_WT_ (lane 6) and GFP-Cα1_Mut_ (lanes 2–5). Molecular mass and C subunit identity is shown at the left and right, respectively. **B.** Analysis of PKA type I holoenzyme formation (RIα-Rluc) with GFP-Cα1_WT_ or GFP-Cα1_Mut_. Expression of Rluc alone defines the background signal (lane 1). 48 h post transfection the luciferase substrate was added and light emission from GFP^2^ and luciferase was monitored in the absence (−) or presence (+) of 50 µM forskolin (fsk) and 100 µM IBMX (+). The results are shown as mean values ± SEM of 3 experiments. C. Analysis of PKA type II holoenzyme formation (RIIα-Rluc) was done as described for PKA type I in panel B.

Cα2_Gly186Val_ was purified by affinity chromatography with RIIα in which Cα_Gly186Val_ was released by addition of cAMP. This suggests that mutation of Cα1 at residue 186 does not interfere with RII association or cAMP activation. However, ATP is required for association between C and RI [53], [54] and given the fact that Cα2_Gly186Val_ probably does not bind ATP efficiently, it may be suggested that Cα_Gly186Val_ will only associate with RII and not RI. To investigate this we determined the binding of Cα1_Gly186Val_ to RIα and RIIα using a Bioluminescence resonance energy transfer (BRET) assay. In these experiments, holoenzyme formation was investigated for all four Cα_Mut_ proteins to test if any of the mutations would influence R subunit binding and cAMP sensitivity. COS-7 cells were cotransfected with either Cα1_WT_ or Cα1_Mut_ N-terminally coupled to Green Fluorescent Protein (GFP), GFP-Cα1_WT_ and GFP-Cα1_Mut_, respectively, and RIα and RIIα C-terminally coupled to *Renilla* luciferase (Rluc),RIα-Rluc and RIIα-Rluc, respectively. Immunoblotting shows the expression of both GFP-Cα1_WT_ and GFP-Cα1_Mut_ (Fig. 4A, lanes 2 to 6). Figure 4, lane 1 depicts the expression of endogenous C subunit in cells transfected with Rluc alone serving as background. In the case of holoenzyme formation with RIα subunits, measurements were normalized by setting the BRET signal in holoenzyme with GFP-Cα1_WT_ to 100% (Fig. 4B, WT “−"). Increasing the intracellular concentration of cAMP with forskolin (fsk) and IBMX (“+") leads to the dissociation of the holoenzyme complex and the reduction of the BRET signal by approximately 50% as reported previously [55]. The same was true for holoenzymes formed with RIα-Rluc and GFP-Cα1_Arg45Gln_, GFP-Cα1_Ser109Pro_, and GFP-Cα1_Ser263Cys_, respectively (Fig. 4B, “+"). In contrast, the GFP-Cα1_Gly186Val_ showed only residual binding to RIα in resting cells (Fig. 4B, Gly186Val “−"), and only marginal activation was detected after stimulation with fsk and IBMX (Fig. 4B, Gly186Val “+").

Analogous to RIα-Rluc measurements with RIIα-Rluc were performed setting unstimulated WT holoenzyme to 100% (Fig. 4C, WT “−"). PKA type II holoenzymes formed with GFP-Cα_WT_ and GFP-Cα_Mut_ dissociate almost completely resulting in a stronger BRET signal reduction (Fig. 4C, “+"). Interestingly, whereas GFP-Cα1_Gly186Val_ does not stably interact with RIα-Rluc, the holoenzymes formed with RIIα-Rluc shows a comparable magnitude to GFP-Cα1_WT_ (Fig. 4C, Gly186Val “−" and WT “−").

## Discussion

Here we have examined the human *PRKACA* gene for mutations by sequencing genomic DNA from 498 Norwegian individuals and by searching for earlier reported mutations in publicly available databases. By genomic sequencing, we identified two nucleotide changes that resulted in the mutations Arg45Gln and Ser109Pro in the Cα1 protein. In public databases we identified two interesting mutations that would lead to residue switches, Gly186Val and Ser263Cys.

Two out of the four mutations (Cα1_Ser109Pro_ and Cα1_Gly186Val_) resulted in significantly reduced kinase activity. It should, however, be noted that comparing the exact activities of Cα1_WT_ and Cα1_Mut_ was a challenge due to differences in transfection efficiencies. We compensated for this by adjusting activities to immunoreactivity in the immunoblots. This demonstrated reduced kinase activity for Cα1_Ser109Pro_, whereas Cα1_Gly186Val_ was kinase inactive. As Gly_186_ is absolutely conserved (Supplementary Fig. S1) and is part of the DFG motif [27] we investigated the mutation affecting this residue in more detail. The importance of Gly_186_ conservation was underscored since mutation was associated with complete abrogation of kinase activity. The exact mechanism that leads to complete inactivation in this case is not known. However, Cα_Gly186Val_ was successfully purified by RIIα-affinity chromatography followed by elution with cAMP. This, together with the fact that Cα_Gly186Val_ forms cAMP-sensitive holoenzymes with RIIα *in vivo* suggests that lack of enzymatic activity is not caused by an overall misfolding of the protein. We rather suggest that lack of kinase activity is due to less extensive molecular changes, affecting only parts of the protein structure. The introduction of a bulky residue in place of Gly_186_ is likely to affect many critical factors necessary for kinase activity. The most apparent explanation is that the aliphatic Val_186_ side chain leads to less efficient binding of Mg1/Mn1 due to displacement of the cation itself or water molecules that are solvating Mg1/Mn1. Loss of Mg1/Mn1 would in turn disable ATP binding, thus explaining the kinase inactivity. This hypothesis is supported by our BRET results, which show exclusive binding to RII but not RI subunits. Moreover, we were unable to purify Cα_Gly186Val_ using PKI affinity chromatography (results not shown). It has previously been shown that the RI subunits, as well as PKI, need ATP to bind C with high affinity [53], [54].

According to our model, it is not unlikely that the hydrogen bond between residues 184 and 186 fails to form in Cα_Gly186Val_, leading to displacement of Asp_184_ and inability to position Mg1 or Mn1 and thereby ATP in the active site. The inability to bind ATP implicates that the C-spine is not established, which is necessary for the active conformation. An alternative explanation for the inactivity is that Val_186_, instead of suppressing binding of Mg1/Mn1 and ATP, is rather displaced, leading to a conformational change in the DFG motif itself. This would lead to a malformed R-spine, which is also thought to be incompatible with kinase activity [26].

Reduced kinase activity was also observed for Cα1_Ser109Pro_. Ser_109_ is located in the middle of β-strand 4 in the small lobe and this β-strand stabilizes the N-terminal end through targeting Ser_109_ to Thr_37_ [48]. It is well known that the N-terminal tail is important for C subunit stability, and deletion of the N-terminus has previously been demonstrated to lead to a significant reduction in thermal stability [56]. We speculate that mutation of Ser_109_ to Pro leads to partial loss of N terminal structure and destabilization of the kinase, and that this may in part provide an explanation for the reduced kinase activity. A second consequence of mutating Ser_109_ may be associated with our recent findings that Ser_109_ belongs to a series of signature residues that can be used to distinguish the Cα from Cβ ortholog (unpublished results). Due to this, the Ser109Pro mutation may result in alteration of Cα-specific functions which do not include holoenzyme formation since our BRET-results showed that Cα1_Ser109Pro_ formed holoenzymes with both RIα and RIIα with comparable affinities as the Cα1_WT_. Full comprehension of the reduced kinase activity and other features associated with mutation at Ser_109_ merits further investigation.

Neither mutations of Arg45Gln nor Ser263Cys influenced apparent kinase activity. Arg_45_ is located near a recently identified conserved pocket in the N lobe known as the N lobe cap, which is above the crucial amino acids Ala_70_ and Lys_72_ [48]. Ala_70_ is part of the C-spine while Lys_72_ is directly involved in ATP binding. It might have been expected that mutation of Arg_45_ would affect kinase activity, also because this residue is highly conserved in metazoan PKA Cα/Cβ homologs (Supplementary Fig. S1) and is clearly under strong purifying selection. The fact that we did not observe any change in phosphotransferase activity suggests that the mutation does not influence the positioning of Ala_70_ and Lys_72_. Despite of this, it may be that mutation of residue 45 affects other features necessary for function of the kinase. It could be speculated that the N lobe cap may also be a docking site for proteins inhibiting kinase activity by disturbing either the C-spine or Lys_72_. This may indicate that the Arg45Gln mutation could be involved in deregulated PKA activity due to altered interactions with so far unidentified interaction partners docking to the N lobe cap. This binding partner does not include the R subunit since the BRET experiments of Cα1_Arg45Gln_ showed no differences in binding and release upon fsk/IBMX stimulation of RIα and RIIα subunits compared to Cα1_WT_. This is also consistent with the localization of Arg_45_ far from the R subunit docking site.

Ser_263_ is highly conserved among metazoa (Supplementary Fig. S1) and is part of the H helix at the very lower end of the large lobe. This site is close to the R subunit docking sites, and the mutation would not be expected to interfere directly with catalytic activity as was also demonstrated here. Rather it could be speculated that a shift from Ser to Cys could influence the mechanism regulating R-C interaction *in vivo*. However, our BRET experiments showed no difference in holoenzyme formation and dissociation of Cα1_Ser263Cys_ to neither RIα nor RIIα subunits compared to Cα1_WT_. Although no effect of a Ser263Cys mutation in Cα was detected, the high degree of conservation of this residue and strong purifying selection is an indicator of a hereto unknown functional importance of Ser_263_.

Among the mutations identified in the sequencing of the *PRKACA* gene, Arg45Gln and Ser109Pro were identified in one and two samples, respectively. Of the two mutations identified in the database search only the Ser263Cys mutation was identified by two independent submitters. To what extent this indicates anything about the prevalence of the different mutations, remains to be verified. However, Gly in the DFG motif is a relatively frequent site of disease-causing mutation in various protein kinases which is most likely due to kinase inactivation [57]. As described above, homozygote targeting mutation of the *PRKACA* gene in mouse is associated with high pre- and postnatal lethality, most probably due to lack of kinase activity at critical steps in embryonic development [30], [32]. Based on this, it is expected that any mutation affecting Cα activity may lead to a severe phenotype and possibly disease in human. Since the Cα1_Gly186Val_ mutation resulted in catalytic inactivation and partial lack of holoenzyme formation, homozygote mutation for Gly_186_ *in vivo* may functionally be considered a gene KO and hence may be incompatible with normal development and life. The same would most likely be the case for the frame shift mutation detected in exon 8. The fact that Cα_Gly186Val_ exclusively forms holoenzyme with RII subunits suggest that individuals with heterozygote mutation of Gly_186_ may have reduced levels of PKA type I holoenzymes, in addition to harboring type II holoenzymes occupied by inactive C subunits, which can hypothetically cause an unbalance in PKA signaling.

The other investigated mutations are likely more compatible with normal development and may not be associated with disease since neither of them influenced holoenzyme formation and mutation of Ser_109_ only partly reduced catalytic activity. Despite this there is a possibility that homozygote mutation of Ser_109_ may be associated with disease since experiments on mice have demonstrated that reduced C subunit gene expression can lead to spinal neural tube defects [33]. In total, 13 nonsynonymous point mutations in the *PRKACA* gene were identified in the present study. For example the Gly186Val mutation was only identified in a single EST sequence, and no frequency data was available. Hence, the information on the prevalence of the various mutations is therefore limited and a full comprehension of their existence in patients or patient groups remains to be elucidated. Finally, it is also worth mentioning that several thousand regions of the human genome have structural variation in large segments termed Copy Number Variation (CNV) [58]. In this paper they report that three out of 95 individuals were found to have a loss of ∼160 kb which included the whole *PRKACA* gene as well as up- and downstream genes. Due to this it may be speculated that a combination of CNV deletion at the *PRKACA* locus and a heterozygote loss-of-function mutation of Cα could be associated with disease due to severe reduction in C subunit gene dose. To what extent this is a cause of disease remains to be determined.

## Supporting Information

Figure S1 Multiple sequence alignment of human PKA Cα (top row) and homologous sequences from a number of metazoan species for the sequence segments containing the 13 mutations discussed in the present study. Enumeration is according to the human PKA Cα1 splice variant and the mutated residues are highlighted. The sequences were obtained from the NCBI (http://www.ncbi.nlm.nih.gov) and UniProt (http://www.uniprot.org) protein sequence databases with the following identifiers: P17612, P05132, P00517, NP_001003032, Q90WN3, A3KMS9, NP_001003470, P22694, P68181, P05131, XP_867543, XP_422379, Q7ZWV0, Q3ZB92, Q7T374, XP_001175934, XP_002740161, CAG44453, XP_393285, XP_968170, NP_476977.
(TIF)
