# Supplementary figures and images for: Identification and Characterization of Novel Mutations in the Human Gene Encoding the Catalytic Subunit Calpha of Protein Kinase A (PKA)
Source: PLoS One. 2012 Apr 13;7(4):e34838. doi: 10.1371/journal.pone.0034838 (PMC3325940; doi:10.1371/journal.pone.0034838)

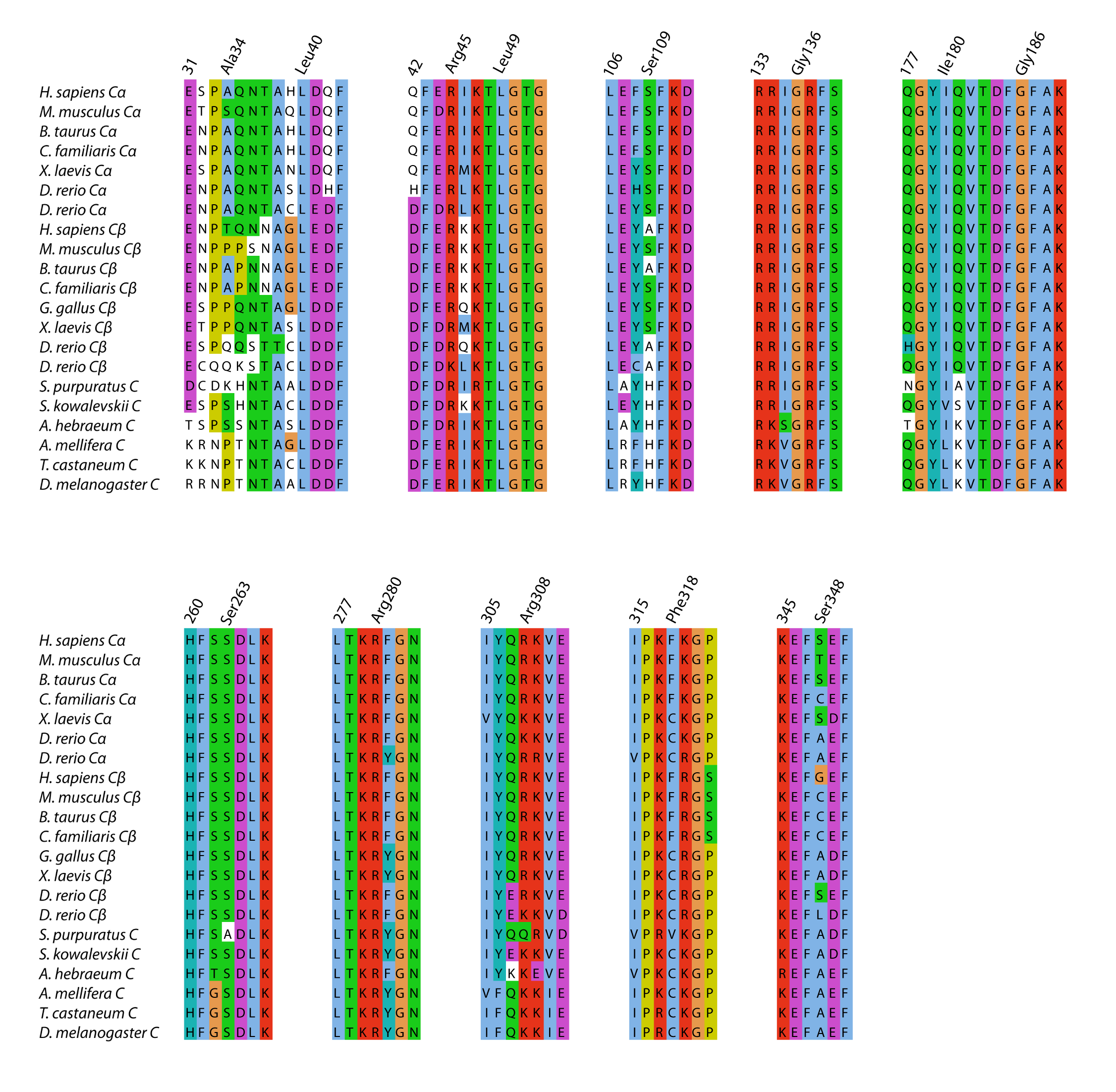

Supplement: Figure S1 — Multiple sequence alignment of human PKA Cα (top row) and homologous sequences from a number of metazoan species for the sequence segments containing the 13 mutations discussed in the present study. Enumeration is according to the human PKA Cα1 splice variant and the mutated residues are highlighted. The sequences were obtained from the NCBI (http://www.ncbi.nlm.nih.gov) and UniProt (http://www.uniprot.org) protein sequence databases with the following identifiers: P17612, P05132, P00517, NP_001003032, Q90WN3, A3KMS9, NP_001003470, P22694, P68181, P05131, XP_867543, XP_422379, Q7ZWV0, Q3ZB92, Q7T374, XP_001175934, XP_002740161, CAG44453, XP_393285, XP_968170, NP_476977. (TIF) [file pone.0034838.s001.tif]
